# Renal Glycosuria as a Novel Early Sign of Colistin-Induced Kidney Damage in Mice

**DOI:** 10.1128/AAC.01650-19

**Published:** 2019-11-21

**Authors:** Sophia L. Samodelov, Michele Visentin, Zhibo Gai, Stephanie Häusler, Gerd A. Kullak-Ublick

**Affiliations:** a Department of Clinical Pharmacology and Toxicology, University Hospital Zurich, University of Zurich, Zurich, Switzerland; b Key Laboratory of Traditional Chinese Medicine for Classical Theory, Ministry of Education, Shandong University, Jinan, China; c Mechanistic Safety, CMO & Patient Safety, Global Drug Development, Novartis Pharma, Basel, Switzerland

**Keywords:** drug-induced kidney injury, colistin, glycosuria, nephrotoxicity, proximal tubule, Kim-1

## Abstract

The polymyxin colistin represents a last-resort antibiotic for multidrug-resistant infections, but its use is limited by the frequent onset of acute drug-induced kidney injury (DIKI). It is essential to closely monitor kidney function prior to and during colistin treatment in order to pinpoint early signs of injury and minimize long-term renal dysfunction. To facilitate this, a mouse model of colistin-induced nephrotoxicity was used to uncover novel early markers of colistin-induced DIKI. Increased urinary levels of kidney injury molecule-1 (Kim-1) as well as glycosuria were observed in colistin-treated mice, where alterations of established clinical markers of acute kidney injury (serum creatinine and albuminuria) and emerging markers such as cystatin C were inaccurate in flagging renal damage as confirmed by histology. A direct interaction of colistin with renal glucose reabsorption was ruled out by a *cis*-inhibition assay in mouse brush border membrane vesicles (BBMV). Immunohistochemical examination and protein quantification by Western blotting showed a marked reduction in the protein amount of sodium-glucose transporter 2 (Sglt2), the main kidney glucose transporter, in renal tissue from colistin-treated mice in comparison to that in control animals. Consistently, BBMV isolated from treated mouse kidneys also showed a reduction in *ex vivo* glucose uptake compared to that in BBMV isolated from control kidneys. These findings support pathology observations of colistin-induced proximal tubule damage at the site of the brush border membrane, where Sglt2 is expressed, and open avenues for the study of glycosuria as a sensitive, specific, and accessible marker of DIKI during colistin therapy.

## Abstract (precis)

The polymyxin colistin represents a last-resort antibiotic for multidrug-resistant infections, but its use is limited by the frequent onset of acute drug-induced kidney injury (DIKI). It is essential to closely monitor kidney function prior to and during colistin treatment in order to pinpoint early signs of injury and minimize long-term renal dysfunction. To facilitate this, a mouse model of colistin-induced nephrotoxicity was used to uncover novel early markers of colistin-induced DIKI.

## INTRODUCTION

Colistin, also known as polymyxin E, is a polypeptide antibiotic used as a last-line treatment option against Gram-negative bacterial infections. Though its use was phased out in the 1970s due to reports of both neuro- and nephrotoxicity and the introduction of other antibiotics, the increase in multidrug-resistant (MDR) infections and the lack of other treatment options has led to renewed interest in colistin in the last decade and a half (1, 2). Because colistin was not subjected to the extensive studies required by contemporary drug regulatory agencies during its development, the bulk of pharmacokinetics and pharmacodynamics information on this drug and derivatives has been accumulated only in recent years (3–6). Likewise, renewed evaluations of the occurrence, severity, and reversibility of renal toxicity have offered a perhaps slightly less catastrophic but still worrisome view (7–10). The most recent study among 249 patients treated with intravenous colistin reported rates of acute kidney injury (AKI), according to the Kidney Disease: Improving Global Outcomes (KDIGO) guideline criteria, as 12% and 29% after 2 and 7 days of treatment, respectively, with 7% of patients requiring renal replacement therapy (11). A retrospective study analyzing the reversibility of colistin-induced renal injury found that 75% of patients that had developed AKI during colistin treatment also progressed to chronic kidney disease (CKD) at a 6-month follow-up compared to 27% in the matched-control subject group (12). This highlights the high risk of long-term renal impairment, particularly in elderly patients, following colistin therapy.

The staple biomarker used in clinics to monitor kidney function is serum creatinine, with estimation of the glomerular filtration rate and monitoring of urine output (13). Large efforts have been put into identifying new markers that more specifically address the etiology of renal insult and identify or predict “subclinical” AKI before serum creatinine elevation rather than assessing renal filtration as an endpoint, with the overall goal of avoiding the irreversibility of ongoing damage. Promising suggested markers arising from such studies include urinary neutrophil gelatinase-associated lipocalin (Ngal) (14) and urinary kidney injury molecule-1 (Kim-1) (15, 16). Ngal is expressed in epithelial cells and has been successfully used in both animal (17) and human (18, 19) studies as a marker for colistin-induced nephrotoxicity. However, it was shown as an unsuitable marker of drug-induced kidney injury (DIKI) during colistin treatment of urinary tract infections in a geriatric patient cohort (20) due to its increased expression upon inflammation and infection (21). Urinary Kim-1 levels have been assessed under colistin treatment in rats, with the report that it is a specific marker of proximal tubular damage (22), with very low expression in healthy kidney tissue (23, 24). Histology is the ideal gold standard for assessing tubular injury, although not always feasible, with Kim-1 representing an example where histological examination and urinary output of the marker have been shown to correlate closely both in rodents and humans (25, 26).

In this study, a mouse model of colistin-induced nephrotoxicity was established to monitor typical and emerging biomarkers of AKI and to identify the earliest markers of DIKI, with physiological relevance in regard to the renal injury caused by this antibiotic. Mice treated intraperitoneally with colistin sulfate once daily for 7 days showed no elevation in most of the biomarkers of kidney injury assessed but a marked increase in urinary Kim-1 as well as glycosuria with comparable diagnostic ability, suggesting urinary glucose as a novel sensitive and specific inexpensive biomarker for colistin-induced nephrotoxicity.

## RESULTS

### Evaluation of clinical kidney injury markers in serum and urine.

**FIG 1:**
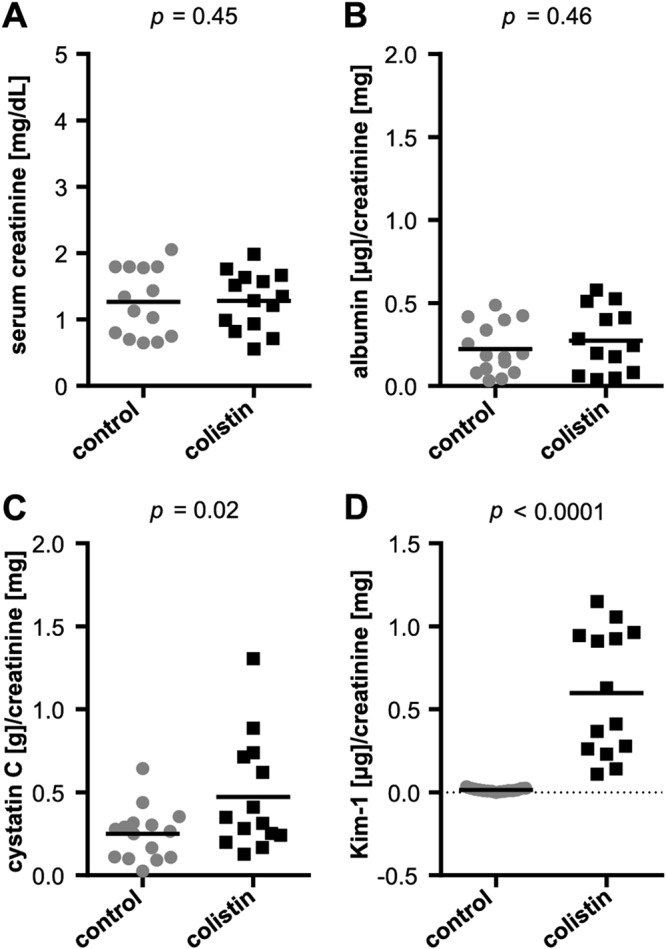
Clinically used markers of DIKI under colistin treatment. Serum creatinine (A), urinary albumin (B), urinary cystatin C (C), and urinary Kim-1 (D) measured in control and colistin-treated mice. Urinary albumin, cystatin C, and Kim-1 were normalized to urinary creatinine. *n* = 15, two-tailed *P* values are indicated, unpaired *t* tests.

Serum samples collected from mice 24 h after the last of seven intraperitoneal (i.p.) injections of 20 mg/kg colistin or the vehicle phosphate-buffered saline (PBS) as a control were first analyzed for creatinine content, the standard marker currently used to assess AKI. Results showed no differences in creatinine between treatment groups (Fig. 1A). Urinary albumin (proteinuria) likewise did not differ between groups (Fig. 1B). Some colistin-treated mice showed elevated urinary cystatin C (CysC) (Fig. 1C), although the majority did not. In contrast, a significant, general increase in urinary Kim-1 in colistin-treated mice was noted (Fig. 1D), indicating tubular injury. As serum creatinine did not fluctuate significantly between mice and treatment groups, reflecting a comparable glomerular filtration, urinary creatinine values could be used to normalize the urinary values measured for other biomarkers (27).

### Histological examination of tubular injury and markers Ngal and Kim-1.

**FIG 2:**
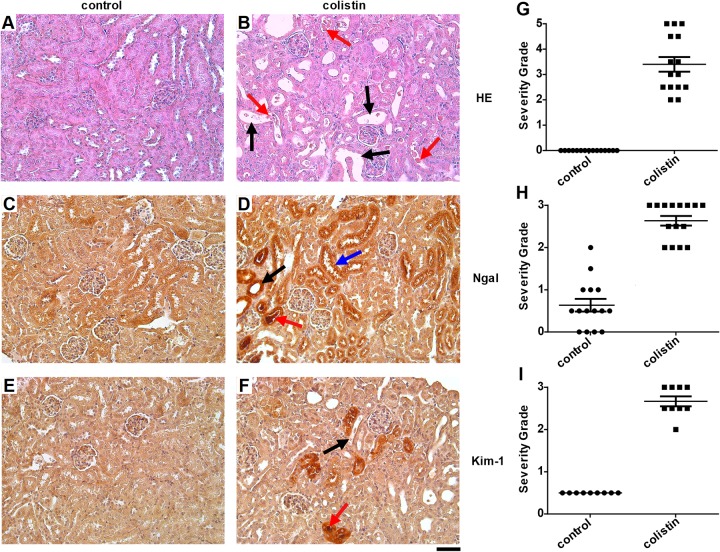
Histological signs of DIKI induced by colistin. (A and B) Hematoxylin and eosin Y (HE) stainings of mouse kidney sections from the control group and colistin-treated mice. (C and D) Ngal stainings. (E and F) Kim-1 stainings. Brown areas indicate positive staining. Black arrows, tubular vacuolization and loss of brush border membrane in proximal tubules; red arrows, tubular protein casts; blue arrow, area of staining with intact brush border membrane; ×20 magnification; bar, 50 μm. (G to I) Severity of AKI was assessed on a severity scale of 0 to 5, based on (i) the presence and amount of protein casts, (ii) presence and percent area with tubular dilation, and (iii) loss and area lacking clear brush border membrane structure. Histology severity grading of samples on a scale of 0 (no staining), 1 (mild), 2 (moderate), and 3 (intense), based on the intensity and area stained per sample. *n* = 15 (G and H), *n* = 9 (I).

Despite the lack of elevation in serum creatinine and urinary albumin, with only some colistin-treated mice showing elevations in urinary CysC upon initial analysis, histological examination of kidney tissue from these mice showed clear signs of kidney damage in all individuals, including tubule dilation, loss of the brush boarder membranes in the proximal tubules, and the formation of protein casts throughout the kidney tissue (Fig. 2A, B, and G). Further histological examination for AKI markers Ngal and Kim-1 showed increased amounts of both proteins in kidney sections from treated mice compared to those in control mice (Fig. 2C to F, H, and I).

### Urinary glucose as a marker of proximal tubule injury upon colistin treatment.

**FIG 3:**
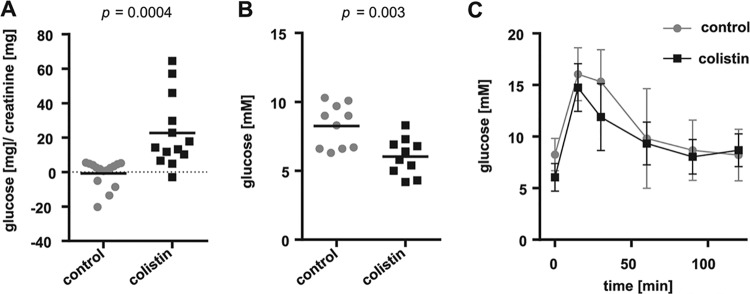
Urinary and systemic blood glucose in mice treated with colistin. (A) Urinary glucose quantified in urine samples collected from each mouse after the final i.p. injection of a 7-day treatment course, normalized to urinary creatinine. *n* ≥ 13, two-tailed *P* value is indicated, unpaired *t* test. (B) Blood glucose values measured in tail blood samples from control and colistin-treated mice after overnight starvation. *n* =10, two-tailed *P* value is indicated, unpaired *t* test. (C) Glucose tolerance test of control and colistin-treated mice performed by measuring glucose in tail-tip blood before and after injection of 1 g/kg d-glucose in solution.

The proximal tubule is the site of renal reabsorption of virtually all organic solutes, which consist mainly of amino acids and glucose. Indications for proximal tubule injury in our colistin-treated mice, as well as the elevations in tubular Kim-1 and Ngal, led us to postulate that deficits in the reabsorption of such solutes might also exist, making them potential urinary biomarkers of colistin-induced tubular injury (28). Indeed, animals treated with colistin exhibited significant glycosuria (Fig. 3A). A glucose tolerance test (GTT) was performed after overnight starvation following the last colistin injection to rule out pleiotropic effects on glucose tolerance by colistin treatment. The areas under the curves (AUCs) obtained for each treatment group were not significantly different (*P* = 0.3), although colistin-treated mice had a significantly lower basal blood glucose value after starvation than the control group, likely as a result of increased urinary glucose excretion (Fig. 3B and C).

### Colistin-induced glycosuria is caused by a reduction in the protein level of the sodium-glucose transporter 2.

**FIG 4:**
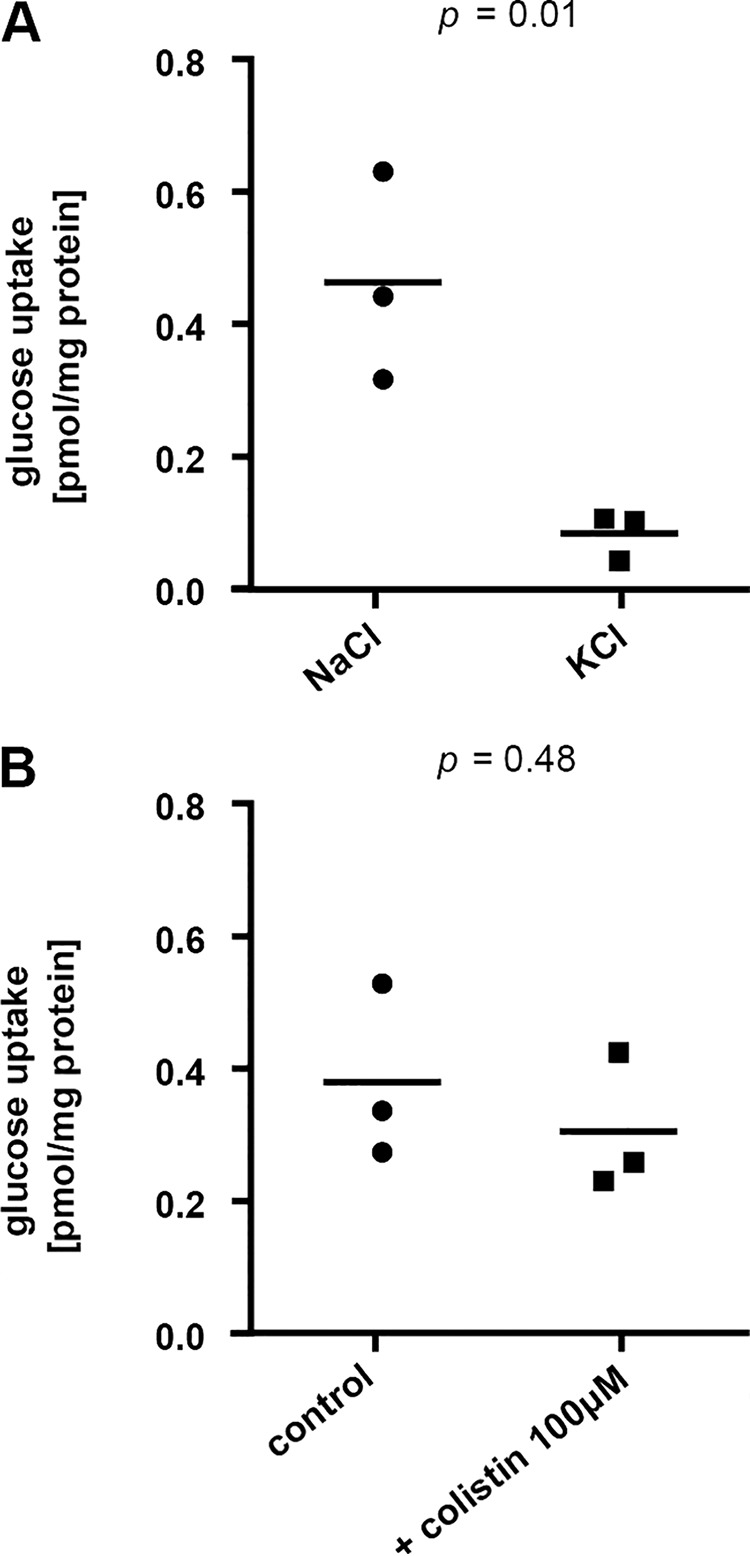
*Ex vivo cis*-inhibition assay. (A) Glucose uptake over 20 s in buffer containing Na^+^ (NaCl) or in Na^+^-free buffer (KCl). (B) Na^+^-dependent uptake of glucose in the presence of colistin sulfate at the indicated concentration. Values were normalized to total protein content, *n* = 3, two-tailed *P* values are indicated, unpaired *t* tests.

Glucose renal reabsorption is a carrier-mediated process that involves two Na^+^-dependent transporters: sodium-glucose transporters 1 (Sglt1) and 2 (Sglt2), with Sglt2 being expressed in segment 1 of the proximal tubule on the brush border membrane and responsible for approximately 97% of renal glucose reabsorption (29). It was previously shown that colistin inhibits l-carnitine and amino acid renal reabsorption by interacting with the zwitterionic/organic cation transporter 2 (OCTN2) and the peptide transporter 2 (PEPT2) (3, 30). To assess the inhibitory effect of colistin on glucose renal reabsorption, a *cis*-inhibition assay was performed with brush border membrane vesicles (BBMV) isolated from untreated mice. As glucose renal reabsorption is a Na^+^-dependent process, uptake of glucose was measured in the presence and absence of Na^+^ to define the Na^+^-dependent transport component. As expected, 90% of glucose uptake was dependent on Na^+^ (Fig. 4A). The Na^+^-dependent uptake of glucose was not reduced in the presence of 100 μM colistin sulfate, a concentration ∼20 times the estimated plasma peak concentration in our experimental setting (31) (Fig. 4B).

**FIG 5:**
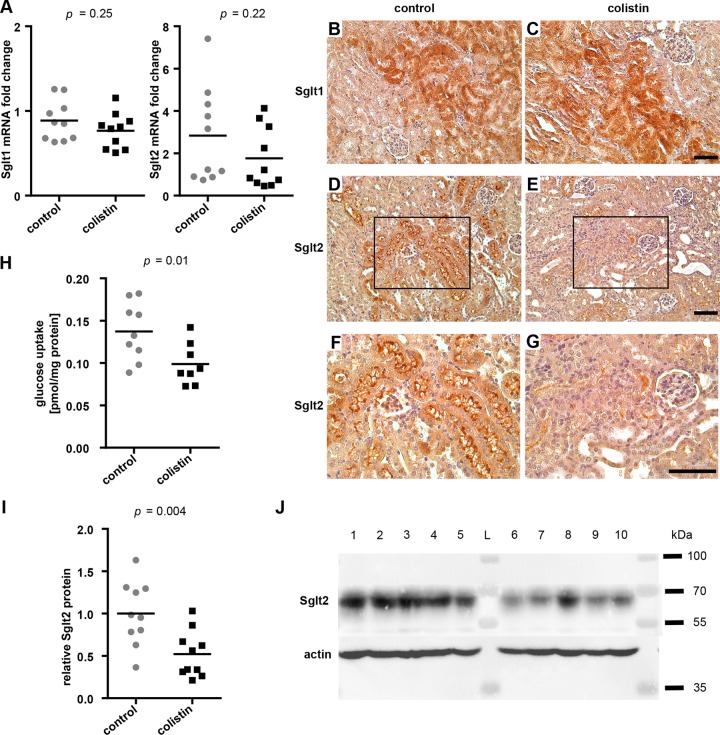
Glucose transporter mRNA, protein, and transport capacity under colistin treatment. (A) mRNA quantification, normalized to villin, of Sglt1 and Sglt2 from whole kidney section homogenates, *n* = 10, two-tailed *P* values are indicated, unpaired *t* tests. (B and C) Sglt1 stainings of mouse kidney sections from control and colistin-treated mice. (D to G) Sglt2 stainings. Brown areas indicate positive staining; ×20 magnification (B to E); ×40 magnification of selected area within the black boxes (F and G); bars, 50 μm. (H) Na^+^-dependent glucose uptake in BBMV isolated from control and colistin-treated mice. Values were subtracted from the respective uptake values measured in the absence of Na^+^ and normalized to total protein content. BBMV from individual mice were assessed separately. *n* ≥ 8, two-tailed *P* value is indicated, unpaired *t* test (I) Quantification of Western blots stained for Sglt2 and pan-actin from two independent experiments, *n* = 10, two-tailed *P* value is indicated, unpaired *t* test. (J) Exemplary Western blot of whole kidney membrane fractions from control (lanes 1 to 5) and colistin-treated (lanes 6 to 10) mice with stainings for Sglt2 and pan-actin, as a loading control. L, protein ladder.

The effect of colistin on the expression of Sglts was then assessed. Transporter transcript levels measured from whole kidney pieces were comparable between the two groups (Fig. 5A). Immunostaining showed no difference in the Sglt1 levels between groups (Fig. 5B and C), whereas the Sglt2 staining intensity and area was markedly reduced in colistin-treated mouse kidney sections (Fig. 5D to G). Western blots of total membrane fractions isolated from the kidneys of individual mice confirmed the reduction in Sglt2 protein amount in colistin-treated mice (Fig. 5I and J). Taken together, colistin treatment does not seem to affect the transcription of glucose transporter Sglt1 or Sglt2 in the kidney under the treatment conditions tested here but leads to a loss of Sglt2 protein, leading to a reduction of renal glucose uptake at the brush border membrane.

Glucose reabsorption capacity after colistin treatment, expected to be reduced by the observed glycosuria and reduction of Sglt2 protein level, was tested *ex vivo* by performing a glucose uptake assay over 20 s in BBMV isolated from control and colistin-treated mice. A significant reduction in the Na^+^-dependent glucose uptake was observed in colistin-treated mice in comparison to that in the control group (Fig. 5H).

### Sensitivity and specificity of urinary biomarkers for early renal damage upon colistin treatment.

**FIG 6:**
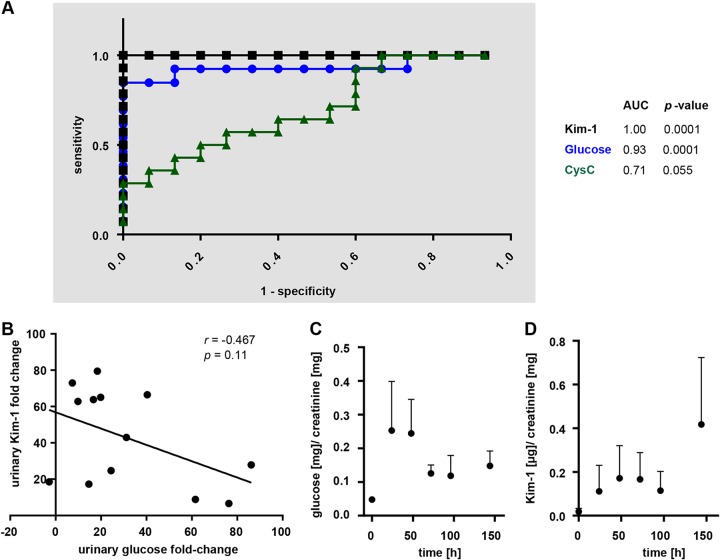
Sensitivity and specificity of urinary markers of colistin-induced renal injury. (A) ROC curves for urinary biomarkers Kim-1 (black), glucose (blue), and cystatin C (green) with AUCs and *P* values indicated, *n* ≥ 13. (B) Urinary Kim-1 fold change from colistin-treated mice plotted against urinary glucose fold change in samples collected after the last treatment. Linear regression line, Pearson’s *r* and *P* value are indicated, *n* = 13. (C and D) Time course of urinary glucose and Kim-1 over the course of treatment in colistin-treated mice. *n* = 5, error bars indicate standard deviations.

In this study, markers that were able to identify or at least partially confirm individual assessments of histologically defined renal injury were the urinary markers CysC, Kim-1, and glucose. Receiver operating characteristic (ROC) curves for these markers, displaying the overall ability of a biomarker to detect a difference between damaged and undamaged kidneys, defined areas under the curves (AUCs) of 0.71 for CysC, 0.93 for glucose, and 1.0 for Kim-1 (Fig. 6A). Data from all treated and untreated mice were used to generate these curves, where hematoxylin and eosin Y (HE) severity grading was used to define damaged and undamaged kidneys. These results indicate that urinary Kim-1 and glucose are both more sensitive and specific for the renal injury incurred under our colistin treatment regime than CysC. Interestingly, urinary Kim-1 and glycosuria had a tendency toward a negative correlation in colistin-treated mice (Fig. 6B). Urine samples were also collected during the course of treatment and analyzed for both glucose and Kim-1 content (Fig. 6C and D). Increases from initial baseline values in colistin-treated mice were observed for both markers as early as 24 h after the first treatment.

## DISCUSSION

The clinically used staple marker for assessing AKI per KDIGO guidelines is serum creatinine. In this study, colistin-treated mice did not exhibit increased serum creatinine (Fig. 1), but histological grading of HE stainings showed clear signs of damage in the kidneys of all colistin-treated animals (Fig. 2). However, other studies performed in mice using similar colistin sulfate doses and treatment durations have indeed shown elevations in serum creatinine (32–34). Differing mouse strain, sex, age, route of administration (intravenous [i.v.] versus i.p.), dosing regimens (twice daily half doses versus once daily full dose), and creatinine quantification method (Jaffe’s method versus an enzymatic method) may all have affected the apparent severity of renal damage in such experiments. Overall, it appears that in this study, renal injury may have been induced to a less severe degree (subclinical) than in past studies, underlining the suitability of our model for assessing the first signs of AKI upon colistin administration. Stressing the fact that recent studies have shown the possible severity and irreversibility of consequences of short-term colistin treatment, it is of great importance to use such models to identify the earliest signs of renal damage that can be used to diagnose the onset of AKI while it is still subclinical (11, 12). The subclinical AKI pattern shown here also grants the advantage of being able to use the individual urinary creatinine values for normalization purposes, correcting for urine concentration and output volume differences, allowing the avoidance of the use of single-housing metabolic cages. However, it is important to stress the concept that this normalization method would not be suitable in any case of clinical AKI, where creatinine excretion is impaired (35–37).

In this study, glycosuria correlated with the renal injury observed upon histological examination. Colistin treatment did not have an effect on overall glucose tolerance yet lowered blood glucose values in starved mice, likely due to the lower glucose reabsorption capacity (Fig. 3). Because pharmacological inhibition of Sglt2-mediated glucose reabsorption effectively reduces blood glucose levels in patients with type 2 diabetes (38), the reduction of Sglt2 protein level at the brush border membrane offers the explanation for the glycosuria observed in these mice (Fig. 5). The comparative marker performance analysis relative to urinary Kim-1 and CysC suggests that glycosuria can be used as an early indicator of subclinical AKI upon colistin administration (Fig. 6). The Kim-1 ROC AUC of 1.0 in this study is comparable to other findings (26, 39), whereas glycosuria shows an AUC of 0.93, showing that both markers have high sensitivity and specificity for colistin-induced AKI. The interesting finding that urinary Kim-1 levels did not correlate with urinary glucose in samples collected after 7 days of colistin treatment suggests that both markers can offer separate and equally relevant information on early renal injury. The time course of urinary Kim-1 values upon renal injury underlie (i) increased expression initiated by renal insult, (ii) cleavage by a metalloproteinase in stressed cells under the control of the mitogen-activated protein (MAP) kinase signaling pathway, and finally, (iii) excretion of the protein in the urine (16, 40, 41). We conclude through this study that glycosuria under colistin treatment is caused by decreased absorptive capacity of the proximal tubule through the decrease of Sglt2 at the brush border membrane. This denotes glycosuria as a possible measure of brush border membrane integrity and function, in regard to glucose reabsorption, whereas urinary Kim-1 may additionally be influenced by other gene regulatory, posttranslational, or cell-stress mediated mechanisms (42). Additional studies would be required to fully understand the reason for the lack of correlation in the increase of these urinary biomarkers under colistin treatment. Nonetheless, glucose and Kim-1 were both detected in the urine concomitantly 24 h after treatment initiation, making glycosuria as early of a marker as Kim-1 is considered for colistin-induced renal injury.

It has been proposed that colistin’s detergent properties may contribute to its nephrotoxicity, which lead to increased permeability of the renal tubular epithelial cell membrane upon protein-mediated reabsorption (endocytosis and facilitative transport) (30, 43–47). Several studies have further suggested that colistin leads to intracellular oxidative stress, particularly at the sites of mitochondria and the endoplasmic reticulum (33, 47–50). Understanding whether the impaired uptake of glucose by proximal tubule cells contributes to the onset and progression of colistin-induced kidney damage is beyond the scope of this work. Nonetheless, it is important to point out that while proximal tubule cells do not rely on glucose as an energy source under physiological conditions, they shift to anaerobic glycolysis to produce the ATP required for cellular regeneration when they experience massive mitochondrial damage (51), as has been documented in the case of colistin-induced kidney injury (49, 52). Thus, the concomitant shortage of free glucose may exacerbate the initial damage caused by colistin. It is interesting to postulate that, particularly, the depletion of glucose, upon the accumulation of colistin in the proximal tubule and induction of intracellular cell stress through mitochondrial damage, may contribute to the high nephrotoxic potential of this drug.

### Conclusions.

The international regulatory agencies have suggested the use of an expanded panel of biomarkers differentially expressed in various regions of the kidney upon insult. These guidelines include CysC, Kim-1, and Ngal among others (53, 54). Ideally, markers of kidney damage used to assess acute kidney injury should be tailored to the medication being administered, within the knowledge available for the respective drug. Most importantly, suggestions and guidelines do not always necessarily hold up in translation, where the accessibility of diagnostics (clinical approval, costs of evaluation, and ease of use) of a biomarker plays a large role in facilitating its regular use in patient care. This study confirms an increase in urinary Kim-1 as a sensitive and specific biomarker of early colistin-induced renal injury and offers glucose as an additional urinary biomarker. Patients receiving such a last-resort therapy often suffer from comorbidities, where the discernment of colistin-induced toxicity from general clinical status increases the complexity of the evaluation of patient data from those receiving colistin. Particularly, patients receiving such a treatment must be assessed before, during, and after treatment due to the increased potential for and the acute occurrence of kidney damage. Glycosuria is not a specific indicator of proximal tubule damage and can also be caused by elevated serum glucose or reduced absorptive capacity of other etiology than discussed here. Thus, some clinical conditions with an underlying glycosuria, such as diabetes, may confound such a readout in a clinical setting. However, we consider it probable that a reduction of Sglt2 in the proximal tubules in diabetic patients under colistin treatment would increase underlying glycosuria in a similar manner as in nondiabetic patients, albeit with higher baseline glucose values. Further studies would be necessary to assess the general applicability and translation of glycosuria as a biomarker of DIKI for colistin-induced nephrotoxicity in human patient subsets, especially toward identifying quantitative cutoff values that maximize its predictive performance. However, the readily available, clinically approved, and low cost of the diagnostic assessment of glycosuria, as well as the sensitivity and specificity of urinary glucose as an early colistin-induced AKI marker, certainly warrant its continued evaluation. We suggest glycosuria monitoring in a clinical setting for patients undergoing colistin treatment as a putative option to justify a more intense screening of the arising expanded panel of biomarkers, where diagnostic options may present as more costly or logistically difficult. This will aid in the identification of patients at risk of severe and long-term renal impairment following colistin therapy.

## MATERIALS AND METHODS

### Mice.

Animal experiments and protocols conformed to the Guide for the Care and Use of Laboratory Animals (U.S. National Institutes of Health) and the Swiss animal protection laws and were approved by the Cantonal Veterinary Office in Zurich, Switzerland (study number 186/2017). Female C57BL/6 mice (Charles River Laboratories, Wilmington, MA) were ordered at 10 weeks of age and acclimatized for 2 weeks before beginning treatment (12 weeks old, 18 to 23 g). Mice were randomly assigned to either colistin or PBS treatment and were housed five mice per cage in individually ventilated cages (IVCs) with free access to food and water. Three separate experiments with a final total of 15 mice per treatment group were performed by two different experimenters for the data obtained in this study. Three additional sets of kidneys from untreated mice of similar age and weight were used for brush border membrane vesicles (BBMV) isolation for the *cis*-inhibition assay. A preliminary experiment was completed with a total of 15 mice and two different colistin sulfate doses (20 mg/kg and 10 mg/kg) to determine the dose sufficient to lead to kidney injury in all treated mice, as confirmed by histology.

### Treatment and sample collection.

Mice were treated with 20 mg/kg colistin sulfate (15,000 U/mg; Sigma-Aldrich, St. Louis, MO) or PBS via i.p. injection for 7 consecutive days. Spontaneous urination samples were collected during handling at various time points during treatment. Not every mouse urinated spontaneously at every handling, leading to a sample size of <15 per treatment group for individual time points. Mice were sacrificed via CO_2_ inhalation 24 h after the last i.p. injection/glucose tolerance test, and serum and kidneys were harvested. Kidney tissue was either fixed in 4% paraformaldehyde for histology, snap-frozen for protein/mRNA quantification, and/or used directly for BBMV isolation for transport assays.

### Glucose tolerance test.

Mice were starved overnight for approximately 14 h. Blood glucose was assessed via tail tip blood sampling using a glucometer (mylife Unio; Ypsomed Distribution AG, Burgdorf, Switzerland) before and after an i.p. injection with a 0.1-g/ml solution of d-glucose (Sigma-Aldrich, St. Louis, MO) at a dose of 1 g/kg.

### Isolation of mouse kidney membrane fractions and immunoblot analysis.

The isolation of a total kidney membrane fraction was modified from a previously described method using one-half of a kidney (55). After homogenization of the tissues with a Polytron in 300 mM sucrose buffer supplemented with a protease inhibitor cocktail (Roche Diagnostics, Indianapolis, IN), the homogenates were centrifuged at 1,300 × *g* in a Sorvall SS34 rotor. The supernatant was centrifuged for 1 h at 100,000 × *g* in a Kontron ultracentrifuge. The total kidney membrane fractions were resuspended in 300 mM sucrose with a 25-gauge needle and stored at −80°C. Protein samples (200 μg) were denatured for 15 min at 65°C, resolved on 8% (wt/vol) polyacrylamide gels, and electroblotted onto nitrocellulose membranes (GE HealthCare, Piscataway, NJ). The membranes were blocked with 5% nonfat dry milk in PBS supplemented with 0.1% (vol/vol) Tween 20 (PBS-T) and then incubated at 4°C overnight with a rabbit anti-Sglt2 antibody, followed by probing with a horseradish peroxidase-conjugated anti-rabbit secondary antibody. Blots were developed with SuperSignal West Femto Maximum Sensitivity Substrate (Thermo Scientific, Waltham, MA) and Fusion FX7 (Vilber Lourmat, Eberhardzell, Germany). As a loading control, the sample blots were stripped and reprobed with anti-pan-actin (NeoMarkers, Fremont, CA).

### Isolation of brush border membrane vesicles.

For the *cis*-inhibition assay, brush border membrane vesicles (BBMV) isolated from both kidneys of one untreated female C57BL/6 mouse were pooled. To assess, *ex vivo*, the renal reabsorption of glucose after 1-week treatment with colistin, BBMV were isolated always from the left kidney of treated mice for individual assessments. BBMV were isolated by a Mg^2+^ precipitation method previously described (56). Excised kidneys were placed in ice-cold isolation buffer (300 mM d-mannitol, 5 mM EGTA, 12 mM Tris-HCl, pH 7.1), homogenized with a Polytron and then mixed with MgCl_2_ to a final concentration of 12 mM. After centrifugation at 3,000 × *g*, the supernatant was centrifuged at 16,000 × *g*, resuspended in vesicle buffer (300 mM d-mannitol, 20 mM HEPES, adjusted to pH 7.4 with Tris) using a glass-Teflon potter, and then spun down again at 16,000 × *g*. Finally, the BBMV pellet was resuspended in vesicle buffer and used directly for the uptake assay and bicinchoninic acid (BCA) assay for protein determination (Interchim, Montluçon Cedex, France).

### BBMV transport assay.

Uptake of glucose was measured over a 20-s interval using a rapid filtration technique as previously described (57). BBMV were preincubated at 37°C, and uptake was initiated by injecting vesicle buffer supplemented with 100 mM NaCl and 100 μM d-glucose and spiked with 5 μCi/ml [^3^H]d-glucose {glucose, D-[6-^3^H(N)], specific activity 45.9 Ci/mmol; PerkinElmer, Switzerland}. Uptake was measured when NaCl was replaced by an equimolar solution of KCl to determine the Na^+^-dependent uptake. Transport was stopped by adding 3 ml of ice-cold stop solution (300 mM d-glucose, 150 mM NaCl, 5 mM Tris-HCl, pH 7.4) followed by filtration through 0.45-μm nitrocellulose acetate filter (Sartorius, Göttingen, Germany). Afterwards, the filter was extensively washed with ice-cold stop solution and dissolved in liquid scintillation fluid (Filter Count; PerkinElmer, Switzerland) to measure radioactivity. Uptake is expressed as picomoles of substrate per milligram of protein. For the *cis*-inhibition assay, glucose uptake was assessed as described above in the presence or absence of colistin sulfate.

### Histology.

Five-micron kidney sections were processed for staining using microwave-based antigen retrieval with citrate buffer (Agilent Dako, Santa Clara, CA). Primary antibodies were Kim-1 (NBP1-76701, 3809-0601A; Novus Biologicals, Centennial, CO), Ngal (ab63929; Abcam, Cambridge, UK), Sglt1 (ab14686, GR306587-14; Abcam, Cambridge, UK), and Sglt2 (Hermann Koepsell, University of Würzburg). Horseradish peroxidase conjugation and detection were performed using the EnVision+ Dual Link System-HRP DAB+ (Agilent Dako, Santa Clara, CA). Samples were immediately counterstained with Mayer’s hematoxylin solution (3870; Biosystems Switzerland AG, Muttenz, Switzerland). For hematoxylin and eosin Y (HE) stainings, sections were stained with Mayer’s hematoxylin and counterstained with eosin Y solution (Sigma-Aldrich, St. Louis, MO). Histological grading of immunostained samples on a scale of 0 (no staining), 1 (mild), 2 (moderate), and 3 (intense) was conducted by two independent scientists in a blinded manner, based on the intensity and area stained per sample. Severity of AKI was determined in HE-stained samples likewise by two independent scientists on a scale of 0 to 5, with 0 being no AKI and 5 the most severe damage, based on (i) the presence and amount of protein casts throughout the kidney section, (ii) presence and percent area with tubular dilation throughout the cortex, and (iii) absence and area without a visible brush border membrane in proximal tubules. The entire kidney section was analyzed for both analyses, and a final score was obtained by averaging the two independently assigned values. Representative images were chosen for each figure, with a severity grade of 0 for control images and severity grades of 3 for immunohistochemistry and 5 for HE stainings for colistin-treated mice.

### Determination of serum and urinary markers of kidney injury.

Urinary glucose and serum and urinary creatinine were measured using the colorimetric/fluorometric kits ab65333 (Abcam, Cambridge, UK) and K625 (Biovision, Milpitas, CA), respectively. Urinary cystatin C (ab201280; Abcam, Cambridge, UK), albumin (ab108792; Abcam, Cambridge, UK), and Kim-1 (ab213477; Abcam, Cambridge, UK) were measured by enzyme-linked immunosorbent assay (ELISA).

### mRNA quantification.

Total RNA was extracted using TRIzol (Ambion by Thermo Scientific, Rockford, IL) from one-third of the snap-frozen left kidney that was thawed on ice and minced prior to resuspension in TRIzol. Two micrograms of total RNA was reverse transcribed with random primers and Superscript II enzyme (Invitrogen, Carlsbad, CA). Real-time PCR analysis using the generated first-strand cDNA was completed using the TaqMan system (Applied Biosystems, Foster City, CA). TaqMan hydrolysis probes for Sglt1 (Mm00451203_m1), Sglt2 (Mm00453831_m1), and villin (Mm00494156_m1) were used.

### Statistical analyses.

All calculations and statistical analyses were performed using GraphPad Prism version 5.
